# Transactions of the Kentucky Medical Society

**Published:** 1853-03

**Authors:** 


### TRANSACTIONS OF THE KENTUCKY MEDICAL SOCIETY.

We have already spoken of the interest of the meeting of the Kentueky Medical Society, which was held, last October, in this city, and of the excellence of several of the reports read at the meeting. The Transactions are now published, and form a neat volume, of wnich we take pleasure in saying, that it reflects honor upon the medical profession in Kentucky. In point of industrious, careful research, sound judgment, and good taste, most of its articles will compare favorably with any to which the various medical associations of our country have given birth. One of the reports is presented to our readers in the current number, in the department of Selections, and there is no need that we should say any thing as to its merits. Professor Miller is high authority on all subjects connected with obstetrical medicine, and in reference to the matters of which his report treats, we suppose, he has had more experience than any other accoucheur in America. It will interest every reader.

The address of the President, Dr. W. L. Sutton, is a plain, sensible, straightforward paper, containing many just reflections on the duty of physicians to their profession, and to the community. He admits that the profession is not as perfect as it might be, but asks the pertinent question, "What have our fault-finding brethren done to improve its condition?" and then proceeds to answer it thus:

A large majority never took a journal: and perhaps nineteen in twenty never contributed an article to a journal in their lives. They surely might have some charity for the puerilities of others, until they have demonstrated that *they* can do better. Jonrnals must be supported by subscriptions, and by contributions. The subscription list is small: and the contribution list vastly more so. I have looked over the Western Journal of Medicine and Surgery, and find that during the year 1851, *ten* gentlemen in Kentucky, besides the Editor, contributed to its pages! Why is this? Why do not the physicians of Kentucky give to the world the results of their observation? Say that an equal number contributed to the Transylvania Jonrnal, and we have *twenty* for the whole State! I repeat, why is this? The excuse ever ready is, 'the press of professional engagements,' 'entirely too busy,' 'have not the time!' Gentlemen, it will not do. Make your excuse 'want of disposition,' 'want of ability,' or the want of any thing but 'time.' In the name of God do not slander our country by saying that of 1470 physicians in the State, only twenty have time to contribute something to a journal annually. We all have our 'anomalous cases,' and our 'astonishing cures,' which we love to rehearse in the ears of our kind and credulous friends; why not lay them before those whose judgment is worth something: whose good opinion will confer honor?

Dr. Chipley's report on the Vital Statistics of Kentucky, is instructive and able. It is accompanied by a map of Kentucky, so colored as to exhibit, at a glance, the relative mortality of different portions of the State:

The counties are arranged in four classes. The first class, colored black, comprises eighteen counties, embracing all those in which the mortality exceeds two per cent. They contain a population of about 55 persons to the square mile—average mortality, one in 41.90 persons, or 2.38 per cent. The highest mortality in any single county was one in 25.72 persons, or 3.88 per cent.

On inspecting the map, the mass of these counties will be found lying in a connected chain from Mercer to Jefferson—six others border on the Ohio river—one on the Mississippi—and two lie isolated amid the more healthy counties. In at least sixteen of these counties a portion of the excessive mortality was probably owing to the prevalence of that fatal scourge of our race—cholera; this may also have been true of the remaining two, but we have no means of ascertaining the fact. It is probable, however, that in ordinary seasons this class comprise the most insalubrious portions of the State, and demand, as we hope they may reeeive withont delay, the severest scrutiny into the causes, with a view to their removal. We are sure that the mortality is far greater than it ought to be, and that judicious sanitary regulations will very materially reduce it.

The second class, lead color, comprise twenty counties, in which the mortality ranged from 1½ to 2 per cent. With a population of about 30 to the square mile, the average mortality was one in 58.92 persons, or 1.69 per cent. Referring to the map, it will appear that all the counties embraced in this class border on the unhealthy counties, or on the Ohio river. A careful consideration of the position of these members of the second class would seem to sustain the opinion that the pestilential causes that produced such excessive mortality in the first class of counties, had a local origin, and that the noxious particles lost their virulence, becoming less concentrated, as they rolled far away from the foyer where they emanated.

The third class, colored blue, comprise thirty counties, embracing all in which the mortality ranged from 1 to 1½ per cent. The population numbers about 29 persons to the square mile; average mortality, one in 79.16 persons, or 1.23 per cent. Five of this class border on the Ohio river; the remainder, it will be perceived, run in more or less connected chains through different portions of the State. A single county of this class lies completely surrounded by those composing the fourth or most highly favored class. This is the only county in the State where the people are wholly thrown upon their own resources in the management of disease—no member of our enterprising profession having yet had the courage to penetrate within its borders. May not the want of medical skill solve the problem, why the mortality here exceeds that of every county by which it is bounded?

The fourth and last class, colored red, comprise the remaining counties of the State, and number 32. The mortality ranges below 1 per cent. They embrace a population of about 21 to the square mile; average mortality, one in 122.35 persons, or 0.81 per cent. The least mortality was one in 376 persons or 0.26 per cent. A few of the counties composing this class are fertile and productive, but the major portion of them are mountainous and sterile—supporting a sturdy but sparse population, living, generally, in the utmost simplicity, being neither enervated by idleness and luxury, nor enfeebled by fashionable habits and dissipation

The Report on Medical Ethics elicited warm approbation from the Society when it was read. It comprises the sentiments of the profession on the points to which it is devoted, and is written with force and energy.

The Report on Surgery, by Prof. Gross, is one of the most elaborate, impartial, and judicious ever submitted to a Medical Society in our country. Dr. Gross has exhausted his subject. Not a surgeon of Kentucky, we believe, from the earliest period down to the day when the Report was read, has been omitted, and equal justice seems to have been rendered to every one who has achieved any thing in this branch of the profession. We shall make copious extracts from this admirable paper in our next number.

Dr. Spilman is entitled to much credit for the care with which he prepared the report on the *Indigenous Botany of Kentucky.*

An abstract of Dr. Darby's Report on Cholera is given, with an interesting account, by Dr. B. P. Drake, of Cholera in Lexington.

Dr. Bell reported the form of a *Case-Book*, which was endorsed by the Society. Dr. Bell will have rendered a great service to the profession if his forcible recommendation of this book shall have the effect of inducing its general adoption and use by his brethren.

